# Efficacy and safety of middle meningeal artery embolisation for chronic subdural haematoma: A systematic review and meta-analysis of randomised controlled trials

**DOI:** 10.1007/s10143-026-04568-4

**Published:** 2026-11-11

**Authors:** Jolie Jin En Wong, Mohammed Jameel, Rocío Pérez, Nader Ameri, Zaid A. Dajani, Nada Alfahad, Guido Firmani

**Affiliations:** 1 Medical student, Cardiff University School of Medicine, Neuadd Meirionnydd, Cardiff, CF14 4YS Wales UK; 2 East Lancashire Hospital Trust, Blackburn, England UK; 3 Clinica Auna, Lima, Peru; 4 Clinica IV Ortoplastica, Instituto Ortopedico Rizzoli, Bologna, Italy; 5 College of Medicine, Imam Abdulrahman Bin Faisal University, Dammam, Saudi Arabia; 6 Barnet Hospital, London, England UK; 7 NESMOS (Neuroscience, Mental Health and Sensory Organs) Department, Faculty of Medicine and Psychology, Sapienza University of Rome, Via di Grottarossa 1035-1039, Rome, 00189 Italy

**Keywords:** Chronic subdural haematoma, Middle meningeal artery embolisation, Recurrence, Randomised controlled trial, Systematic review, Meta-analysis

## Abstract

Chronic subdural haematoma (cSDH) frequently recurs following standard treatment. Middle meningeal artery embolisation (MMAE) may reduce recurrence by decreasing perfusion of the vascularised subdural membranes. This systematic review and meta-analysis evaluated the efficacy and safety of MMAE versus standard management. This PRISMA-compliant systematic review and meta-analysis (PROSPERO: CRD420261325584) searched PubMed, Ovid (MEDLINE and Embase), Web of Science, and the Cochrane Library from inception to 28 September 2026. Randomised controlled trials comparing MMAE-based treatment with standard management in adults with radiologically confirmed cSDH were included. Risk ratios (RRs) with 95% confidence intervals (CIs) were pooled using random-effects models. The primary outcome was study-defined treatment failure. Twelve randomised controlled trials (*n* = 2,429; 1,202 MMAE; 1,227 control) were included in the primary analysis. Treatment failure occurred in 9.1% versus 18.3%, respectively. MMAE reduced treatment failure (RR 0.52, 95% CI 0.39–0.69), corresponding to an absolute risk reduction of approximately 8.8% and an NNT of approximately 12. In subgroup analyses, benefit was observed in both surgery-only comparator trials (RR 0.41, 95% CI 0.23–0.76) and mixed surgical/conservative comparator trials (RR 0.55, 95% CI 0.37–0.81), with no significant subgroup difference (*p* = 0.317). MMAE also reduced reoperation/surgical rescue (RR 0.40, 95% CI 0.31–0.52). All-cause mortality (RR 0.72, 95% CI 0.37–1.43) and severe adverse events (RR 0.83, 95% CI 0.60–1.16) did not differ significantly between groups. Certainty was moderate for treatment failure and reoperation/surgical rescue and low for mortality and severe adverse events. MMAE-based treatment reduces treatment failure in cSDH, with no statistically significant difference in mortality or severe adverse events. Further well-powered randomised trials using standardised efficacy and safety outcomes are needed to define the optimal role of MMAE in cSDH management.

**Supplementary Information:**

The online version contains supplementary material available at https://doi.org/10.1007/s10143-026-04568-4.

## Introduction

Chronic subdural haematoma (cSDH) is one of the most prevalent neurosurgical conditions with an annual incidence ranging from 1.7 to 20.6 per 100,000 persons, a burden projected to rise with global population ageing [1, 2]. Once viewed as a passive blood accumulation from bridging-vein rupture, cSDH is now understood to be driven by persistent inflammation and pathological neoangiogenesis with leaky capillaries within the subdural membranes [3–5]. Remote head trauma is thought to be the most common cause, with risk factors including advanced age, male sex, alcoholism, and anticoagulant or antiplatelet use [6].

This biological activity provides a rationale for treatments targeting the vascular supply and inflammatory activity of subdural membranes, including middle meningeal artery embolisation (MMAE) and pharmacological approaches [7–9]. Surgical intervention aims to remove the haematoma and relieve mass effect [8, 10], most commonly by burr-hole drainage, with mini-craniotomy or craniotomy used selectively [8, 10, 11]. Recurrence remains a major complication, reported in 9–33% of cases [10, 12, 13], and postoperative complications, such as infection, seizures, or haematoma expansion, are particularly relevant in older patients with multimorbidity [10].

MMAE has emerged as a minimally invasive adjunct or alternative to surgery [14], targeting outer membrane feeder vessels to reduce neovasculature and fluid exudation [15]. Indications include high surgical risk, recurrence prevention after burr-hole evacuation, preoperative embolisation to reduce postoperative complications, and, selectively, primary treatment [16]. Recent randomised controlled trials (RCTs) including EMBOLISE [17], STEM [18], and MAGIC-MT [19] have expanded the evidence base, although differences in populations, embolic agents, comparator strategies, and outcome definitions complicate interpretation. Several RCT-focused meta-analyses have synthesised this evidence [20, 21]. This review extends previous syntheses by incorporating three additional 2026 RCTs (EMMA-Can [22], G-MEMBRANE [23], and MEMBRANE [24]), expanding the primary analysis to 12 RCTs (*n* = 2,429); including both surgery-only and mixed surgical/nonsurgical management pathways; and separately pooling reoperation/surgical rescue, alongside GRADE assessments and trial sequential analysis (TSA) of the principal efficacy outcomes.

This systematic review and meta-analysis (SRMA) therefore evaluates randomised evidence comparing MMAE with standard management, focusing on treatment failure, reoperation/surgical rescue, mortality, safety, and other reported clinical outcomes.

## Materials and methods

This systematic review and meta-analysis was conducted in accordance with the Preferred Reporting Items for Systematic Reviews and Meta-Analysis (PRISMA) guidelines [25] and was prospectively registered in PROSPERO (CRD420261325584).

A comprehensive search of PubMed, Ovid (Embase and MEDLINE), Web of Science, and the Cochrane Library was conducted from inception to 28 September 2026 using MeSH terms, free-text terms, and Boolean operators. Search strategies were refined after an initial screening for missing keywords and are provided in Supplementary Appendix 1. Reference lists of included studies and relevant reviews were also searched.

### Eligibility criteria

Eligible studies were RCTs of adults (aged ≥ 18 years) with radiologically confirmed cSDH in which MMAE was performed as part of the treatment strategy. Comparators included standard management such as burr-hole drainage, craniotomy, surgery, and/or conservative approaches. Preprints were eligible if sufficient methodological and outcome data were available, with their influence examined through sensitivity analysis.

Studies were excluded if they lacked random allocation or a direct MMAE-control comparison. Observational, retrospective, registry-based, and non-randomised controlled studies, as well as studies involving paediatric patients, acute SDH, animal models, or non-English publications, were excluded.

### Screening and study selection

Database searches yielded 602 records (PubMed, *n* = 205; Cochrane Library, *n* = 86; Web of Science, *n* = 167; and Ovid MEDLINE/Embase, *n* = 144). After removing 303 duplicates, 299 records were screened, and 58 full-text reports were assessed. Of these, 47 were excluded, and one additional report was identified through citation searching, resulting in 12 included RCTs. Two reviewers (J.W. and N.Al.) independently screened records using Rayyan (version 3.0), with disagreements resolved by consensus or third-reviewer adjudication (G.F.).

### Data extraction

Two reviewers (N.Am. and Z.D.) independently extracted data using a standardised Microsoft Excel form developed by J.W. Variables included study characteristics, population size, intervention, comparator, and outcomes of interest.

### Quality and certainty of evidence evaluation

R.P. and N.Al. independently assessed risk of bias using the Cochrane Risk of Bias (ROB) 2 tool [26], with review by J.W. and disagreements resolved by consensus or consultation with a senior author (G.F.). Certainty of evidence was evaluated using the Grading of Recommendations Assessment, Development, and Evaluation (GRADE) framework by R.P. and reviewed by J.W [27].

### Statistical analysis

Meta-analysis was performed using R (version 4.5.2). Dichotomous outcomes were analysed using risk ratios (RRs), with corresponding 95% confidence intervals (CIs). Random-effects models were used because of anticipated clinical and methodological heterogeneity, with Mantel-Haenszel weighting, Hartung-Knapp adjustment, and restricted maximum likelihood (REML) estimation of τ². Double-zero trials contributed no weight. Heterogeneity was assessed using the Chi-squared test (*p* < 0.10) and I², interpreted as low (25%), moderate (50%), or high (75%).

The primary outcome was study-defined treatment failure, using each trial’s original efficacy failure definition, including recurrence, progression, reoperation, reintervention, or other trial-defined endpoints. These definitions were not retrospectively harmonised and are detailed in Supplementary Table S1. Reoperation or surgical rescue was additionally analysed where comparable binary data were available. Absolute risk estimates, absolute risk reduction (ARR), and the number needed to treat (NNT) were estimated by applying the pooled RR to the pooled control-group event rate.

Secondary outcomes included all-cause mortality, severe adverse events (as defined by each study), functional outcomes (e.g., the modified Rankin Scale), neurological improvement, and radiological measures such as haematoma thickness or volume. Continuous outcomes were extracted as means and standard deviations where available but were synthesised narratively because of heterogeneity in definitions, measurement methods, follow-up, and reporting. Outcome-specific denominators were based on the populations reported by each trial for that outcome and therefore varied between efficacy and safety analyses.

Predefined subgroup analyses compared surgery-only with mixed comparator groups (surgery with or without conservative management). Leave-one-out sensitivity analyses assessed robustness, and prediction intervals were calculated to estimate expected treatment effects across clinical settings. A two-sided p-value of < 0.05 was considered statistically significant. A drapery plot was generated to visualise study-specific and pooled effect estimates across confidence levels (Supplementary Figure S1).

TSA was performed for study-defined treatment failure and reoperation/surgical rescue using the RTSA package (version 0.2.2) in R. Trials were ordered by earliest public availability. Required information size (RIS) was calculated assuming a two-sided α of 5%, 80% power, and 20% anticipated relative risk reduction, using pooled control event rates of 18.3% for treatment failure and 11.6% for reoperation/surgical rescue.

Lan-DeMets O’Brien–Fleming monitoring boundaries were constructed using an alpha-spending function, with corresponding non-binding futility boundaries based on beta spending where these could be defined at the accrued information fraction. The RIS was adjusted for between-study heterogeneity using diversity (D²). For calculation of the cumulative Z-curve, Mantel–Haenszel common-effect statistics were used while the estimated between-study variance (τ²) was zero; when τ² became non-zero, DerSimonian–Laird random-effects estimates with the Hartung–Knapp–Sidik–Jonkman variance adjustment were used.

Crossing the trial sequential monitoring boundary was interpreted as sequential evidence of benefit under the prespecified TSA assumptions. Attainment of the RIS was used to assess whether the prespecified information size had been accrued. TSA-adjusted 95% confidence intervals were also calculated.

### Ethics statement

Ethics approval was not required because this SRMA analysed previously published aggregate data and involved no participation of humans or animals.

## Results

### Study characteristics

Twelve RCTs were included in the primary analysis (*n* = 2,429; 1,202 MMAE; 1,227 control). Study selection is summarised in the PRISMA flow diagram (Supplementary Figure S2).

The included studies evaluated MMAE either as an adjunct to surgery or within treatment pathways allowing both standalone and/or adjunctive use. Eight trials compared MMAE with surgery alone (predominantly burr-hole, twist-drill drainage, with craniotomy permitted where clinically indicated), while four compared MMAE with mixed management pathways incorporating both surgical and/or conservative treatment strategies. In the MAGIC-MT trial [19], approximately 78% of patients underwent burr-hole drainage, whereas STEM [18], Tavakkoli et al. [28] and MEMBRANE [24] permitted surgical or conservative treatment based on clinical indication. Therefore, most available evidence reflects MMAE used alongside surgical management of cSDH.

Follow-up duration ranged from 4 to 6 weeks to 12 months, with most studies reporting outcomes between 3 and 6 months. The primary outcome was study-defined treatment failure, while secondary outcomes included mortality, severe adverse events, functional outcomes, neurological outcomes, and radiological parameters. Detailed characteristics of the included studies are summarised in Table 1. One included trial had not undergone peer review at the time of analysis.

**Table 1:** Characteristics of included studies

Author (Year)	Country	Trial name	Multicentre	Population	Randomised *N* (MMAE/control)	MMAE treatment strategy, *n*	Comparator strategy, *n*	Embolic agent	Age, years (MMAE/control)	Male, % (MMAE/control)	Follow-up	Funding source
Tavakkoli et al. (2025) [28]	USA	NR	No	cSDH managed with surgery or observation	46 (23/23)	Surgery + MMAE: 18; observation + MMAE: 4	Surgery: 21; observation: 2	Predominantly Embospheres/microparticles; coil used in one case	Median 76 (68–83) / 79 (70–86)	82 / 65	12 months	None
Ng et al. (2020) [29]	France	NR	No	Symptomatic cSDH requiring surgery	46 (21/25)	Surgery + adjunct MMAE: 21	Surgery alone: 25	PVA 150–250 μm; coils if required	Mean 77.4 ± 10.9 / 74.7 ± 13.9	52.6 / 59.1	3 months	No funding declared
Lam et al. (2023) [30]	Australia	NR	Yes (2 centres)	Symptomatic cSDH requiring surgical evacuation	36 (17/19)	Surgery + postoperative MMAE: 17 assigned; 16 received MMAE	Surgery alone: 19	Squid-12, Onyx-18, PHIL 25%, n-BCA/Lipiodol	Mean 64.2 / 72.4	75.0 / 57.9	3 months	Life Health Care Group
Debs et al. (2024) [31]	USA	NR	No	Symptomatic cSDH undergoing surgical evacuation	35 (17/18)	Surgery + postoperative MMAE: 17	Surgery alone: 18	Onyx	Mean 66.1 / 70.8	70.6 / 61.1	4–6 weeks	NR
Manzato et al. (2025) [32]	Brazil	NR	No	Symptomatic cSDH requiring surgery	76 haematomas (33/43)	Surgery + postoperative MMAE: 33 haematomas	Surgery alone: 43 haematomas	Histoacryl (n-BCA) + Lipiodol	Mean 73.3 ± 10.9 / 74.1 ± 10.8	84.8 / 74.4	90 days	No specific grant
Fiorella et al. (2025) [18]	Multinational	STEM	Yes (32 centres)	Symptomatic cSDH assigned to surgical or nonsurgical standard treatment before randomisation	310 (149/161)	MMAE + surgery: 91; MMAE + nonsurgical treatment: 58	Surgery: 98; nonsurgical treatment: 63	Squid	Mean 72.8 ± 10.4 / 73.4 ± 11.3	65.1 / 73.9	180 days	Balt USA
Liu et al. (2024) [19]	China	MAGIC-MT	Yes (31 centres)	Symptomatic nonacute SDH managed surgically or nonsurgically	727 (365/362)	Burr-hole + MMAE: 281; nonsurgical + MMAE: 79	Burr-hole drainage: 284; nonsurgical treatment: 78	Onyx	Median 68.5 (60–74) / 70 (61–75)	81.1 / 84.0	90 days	Shanghai Shenkang Hospital Development Center and others
Shotar et al. (2025) [33]	France	EMPROTECT	Yes (12 centres)	cSDH treated surgically; recurrent or high risk of recurrence	342 (171/171)	Surgery + postoperative MMAE: 171	Surgery alone: 171	Embosphere/TAGM 300–500 μm	Median 77 / 77	81.3 / 78.9	6 months	No funding declared
Davies et al. (2024) [17]	USA	EMBOLISE	Yes (39 centres)	Subacute or chronic SDH requiring surgery	400 (197/203)	Surgery + adjunct MMAE: 197	Surgery alone: 203	Onyx	Median 73 / 71	72.6 / 73.4	90 days	Medtronic
Shankar et al. (2026) [22]	Canada	EMMA-Can	Yes (9 centres)	Unilateral symptomatic cSDH undergoing surgical drainage	192 (98/94)	Surgery + postoperative MMAE: 98	Surgery alone: 94	Onyx-18	Mean 71.8 ± 10.6 / 71.9 ± 10.9	74.2 / 72.0	90 days	NR
Goelz et al. (2026) [23]	Germany	G-MEMBRANE	No	cSDH requiring burr-hole evacuation	93 total; arm-level randomised N NR	Burr-hole surgery + MMAE; mITT *n* = 26	Burr-hole surgery alone; mITT *n* = 38	PVA 40–150 μm, Onyx 18/20, or Squid 12/18	Mean 73.7 ± 12.6 / 75.3 ± 13.9	79.2 / 58.3	3 months	German Social Accident Insurance (FR341)
Kellner et al. (2026) [24]	USA/China	MEMBRANE	Yes (30 centres)	Symptomatic cSDH assigned to surgical or nonsurgical SOC before randomisation	376 (188/188)	Surgery + MMAE: 133; NSMM + MMAE: 55	Surgery alone: 132; NSMM alone: 56	TRUFILL n-BCA	Mean 70.9 ± 10.6 / 70.3 ± 12.1	76.1 / 73.9	6 months	Johnson & Johnson MedTech

Randomised N represents the total number of participants randomised in each trial, except for Manzato et al., which randomised haematomas rather than individual participants. Outcome-specific denominators were smaller in some studies because of withdrawal, loss to follow-up, or missing outcome data. In Tavakkoli et al., one participant allocated to MMAE withdrew after randomisation and before treatment. In MAGIC-MT, five participants allocated to MMAE withdrew consent, resulting in an ITT population of 360 MMAE and 362 control participants; treatment-strategy counts shown are based on this ITT population. In EMMA-Can, 192 participants were randomised, with 186 (93/93) included in the primary outcome analysis. In G-MEMBRANE, 93 participants were randomised, and 64 (26 MMAE/38 control) were included in the modified ITT primary analysis. Safety outcomes used each trial’s reported safety (as-treated) population where applicable, so denominators may differ from randomised numbers.

### Primary outcome

#### Study-defined treatment failure

Twelve RCTs were included in the primary analysis (*n* = 2,429; 1,202 MMAE and 1,227 control) [17–19, 22–24, 28–33]. Manzato et al. [32] contributed haematoma-level rather than patient-level data. Treatment failure occurred in 109/1,202 (9.1%) in the MMAE group and 224/1,227 (18.3%) in the control group.

**Fig. 1:**
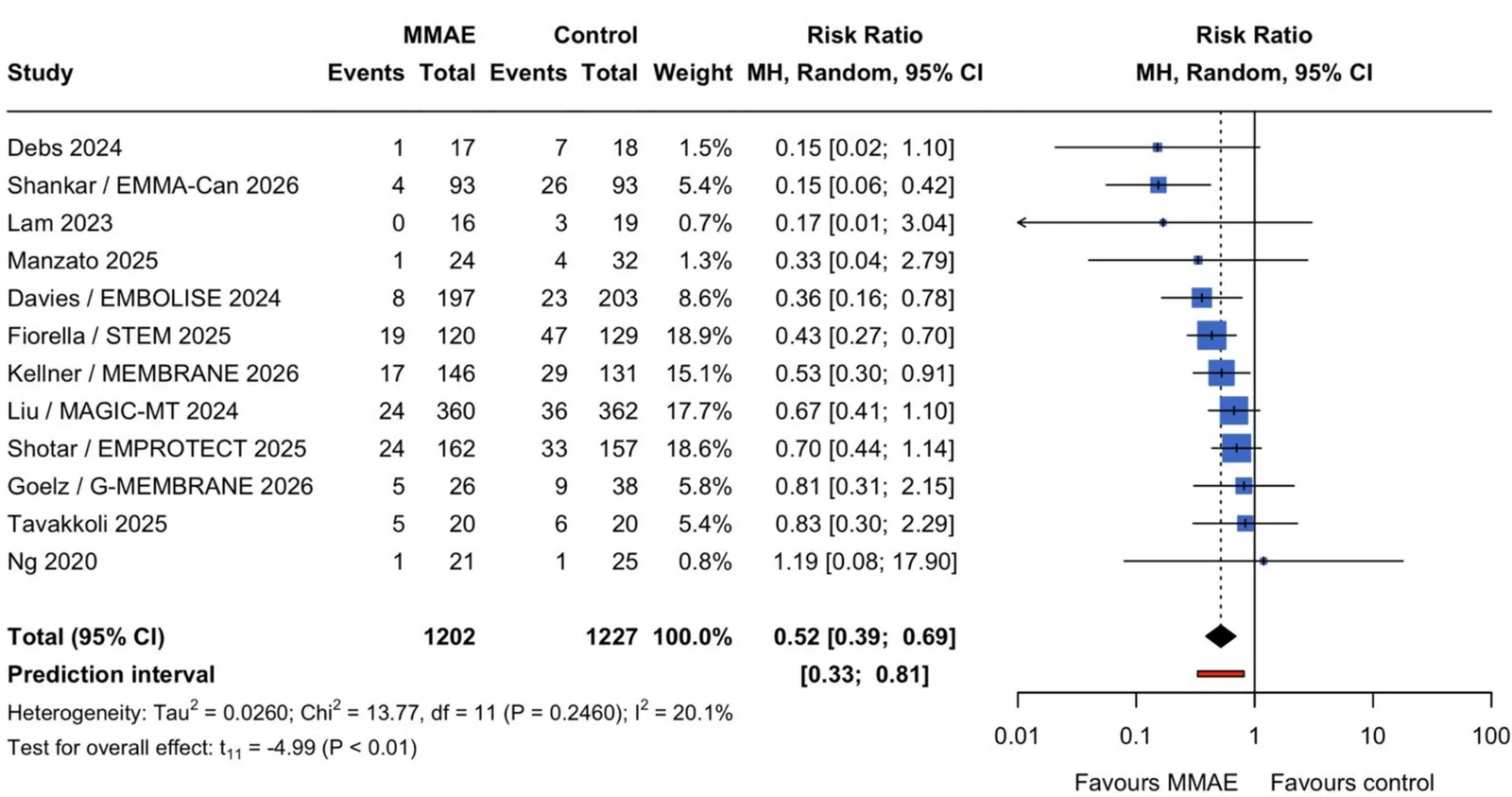
Forest plot of study-defined treatment failure comparing MMAE versus standard management

MMAE significantly reduced treatment failure compared with standard management (RR 0.52, 95% CI 0.39–0.69), corresponding to an ARR of 8.8% and an NNT of approximately 12. Low heterogeneity was observed (I² = 20%). The 95% prediction interval (0.33 to 0.81) remained entirely below unity, suggesting that the effect in a new trial setting would be expected to favour MMAE (Fig. 1).

#### Subgroup analysis by comparator type

**Fig. 2:**
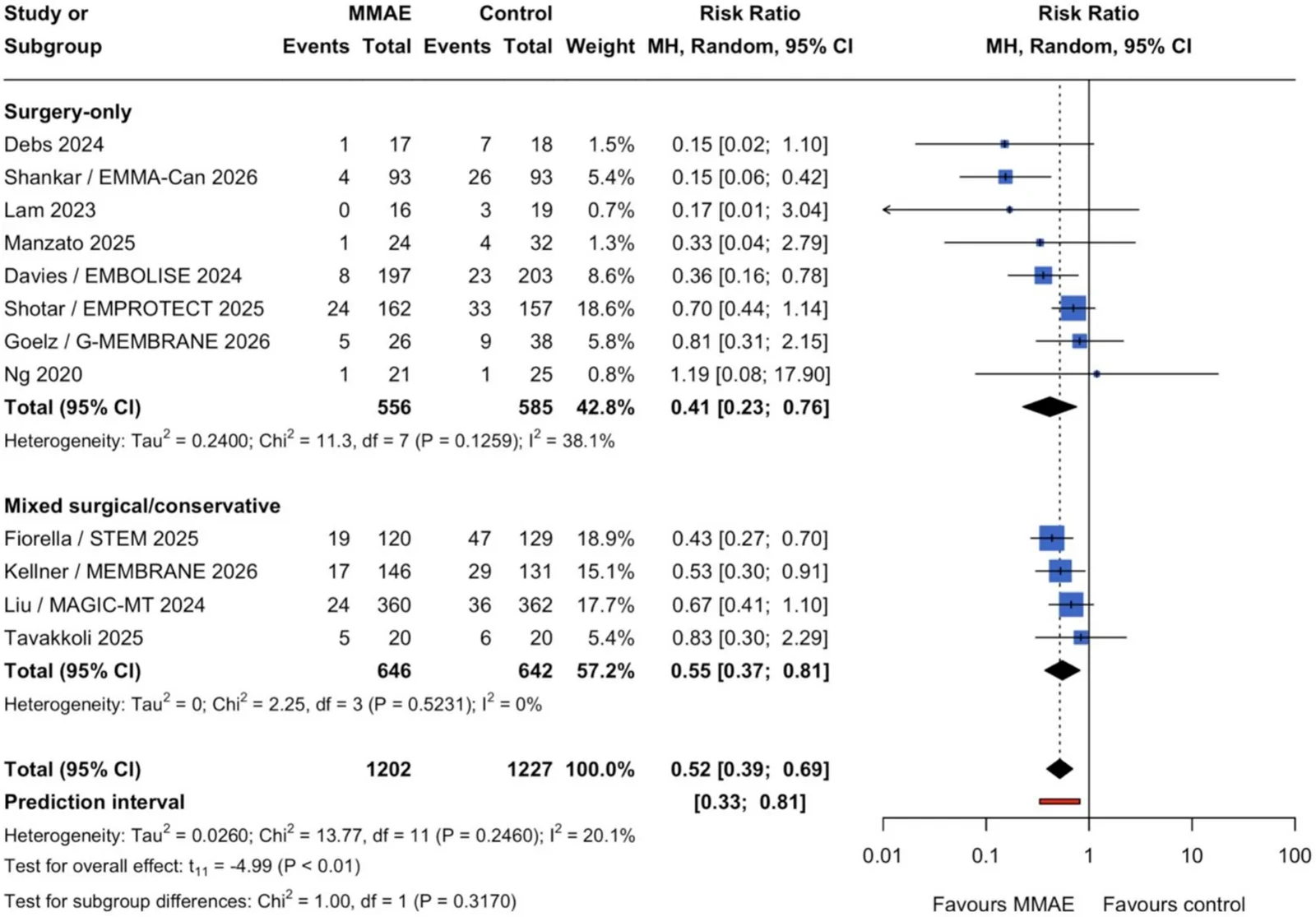
Subgroup analysis of study-defined treatment failure by comparator type (surgery-only vs. mixed surgical/ conservative comparator)

In surgery-only comparator trials, MMAE significantly reduced treatment failure (RR 0.41, 95% CI 0.23–0.76), with moderate heterogeneity (I² = 38.1%) (Fig. 2). In trials using mixed surgical/ conservative comparator strategies, MMAE also significantly reduced treatment failure (RR 0.55, 95% CI 0.37–0.81), with no observed heterogeneity (I² = 0%).

The test for subgroup differences was not statistically significant (*p* = 0.317), indicating no clear evidence that treatment effect differed by comparator type. Therefore, these findings should be considered exploratory and hypothesis-generating.

#### Sensitivity analysis

**Fig. 3:**
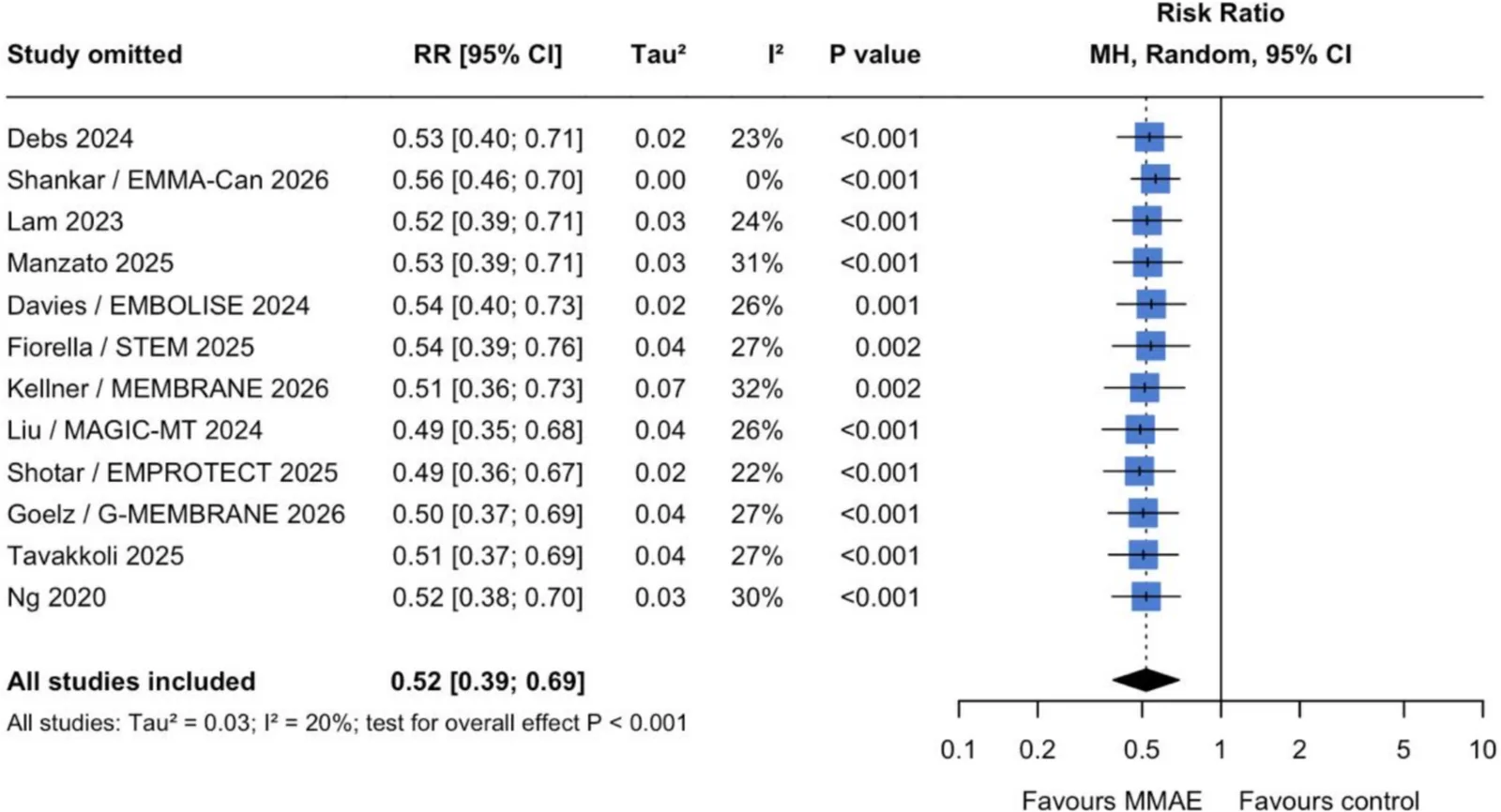
Leave-one-out sensitivity analysis of study-defined treatment failure

Leave-one-out sensitivity analysis showed that the pooled effects consistently favoured MMAE. Exclusion of Tavakkoli et al. [28], which was available as a preprint, did not materially alter the pooled effect estimate (RR 0.51, 95% CI 0.37–0.69). Across all iterations, pooled RRs ranged from 0.49 to 0.56, statistical significance was maintained, and heterogeneity remained low (I² = 0–32%). These findings indicate that the overall treatment effect was not driven by any single study (Fig. 3). A drapery plot demonstrating the distribution of study-specific and pooled effect estimates across a range of confidence levels is provided in Supplementary Figure S1**.**

#### Reoperation/surgical rescue

**Fig. 4:**
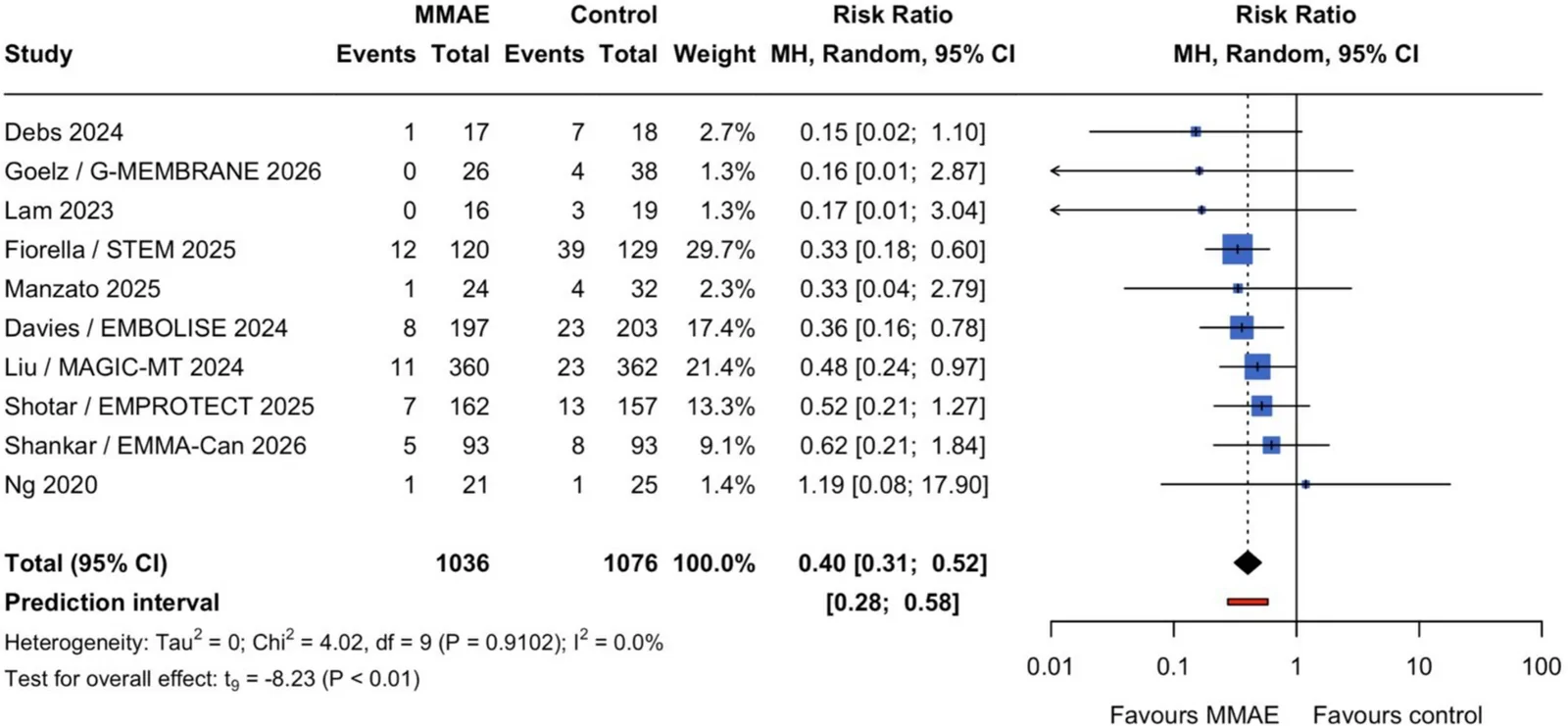
Forest plot of reoperation/surgical rescue comparing MMAE versus standard management

Ten RCTs contributed comparable binary data on reoperation or surgical rescue (*n* = 2,112; 1,036 MMAE and 1,076 control). Events occurred in 46/1,036 (4.4%) in the MMAE group and 125/1,076 (11.6%) in the control group. MMAE significantly reduced the risk of reoperation/surgical rescue compared with standard management (RR 0.40, 95% CI 0.31–0.52) (Fig. 4).

#### Trial sequential analysis

For treatment failure, the diversity-adjusted RIS was 5,221 (D² = 34%), of which 2,429 (47%) had accrued. The Z-curve crossed the monitoring boundary for benefit upon inclusion of EMBOLISE, at 1,487 (Z = − 4.83; boundary − 4.04), and remained beyond it through the remaining six trials (final Z = − 4.90; boundary − 3.14). The TSA-adjusted RR was 0.51 (95% CI 0.34 to 0.79) (Supplementary Figure S3).

For reoperation/surgical rescue, D² was 0%, so the RIS of 5,450 needed no heterogeneity adjustment; 2,112 (39%) had accrued. The curve crossed the benefit boundary at the same trial (Z = − 5.28; boundary − 4.14) and remained beyond it at the final analysis (Z = − 5.71; boundary − 3.50). The TSA-adjusted RR was 0.39 (95% CI 0.22 to 0.69) (Supplementary Figure S4).

In both outcomes, the curves crossed the conventional threshold of Z = − 1.96 by the third trial, before crossing the more stringent sequential monitoring boundaries. The cumulative Z-curves subsequently crossed the sequential monitoring boundaries for benefit before the RIS were reached, providing sequential evidence of benefit under the prespecified TSA assumptions. Nevertheless, the RIS was not reached for either outcome.

### Secondary outcomes

#### Mortality

**Fig. 5:**
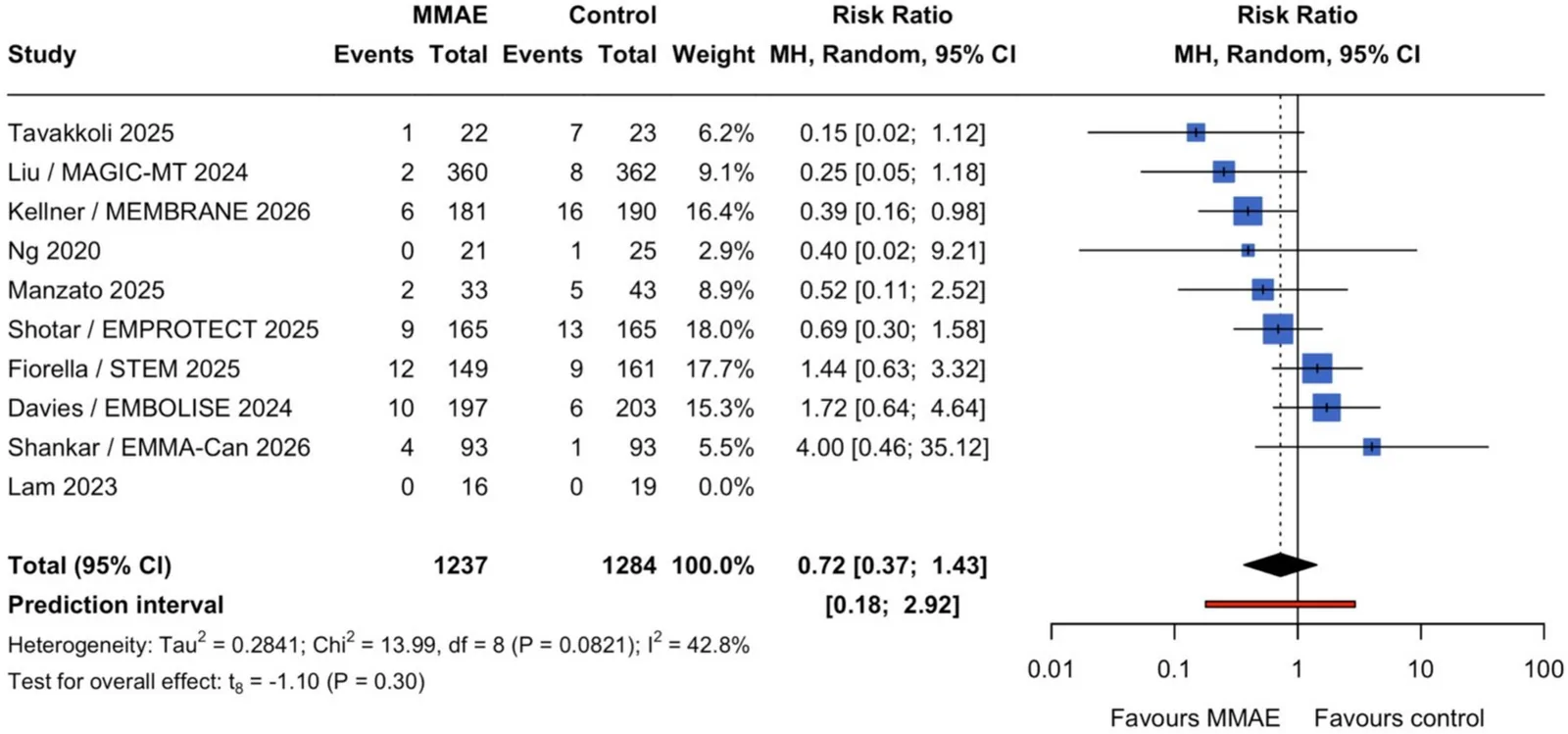
Forest plot of all-cause mortality comparing MMAE versus standard management

Ten RCTs contributed data on all-cause mortality (*n* = 2,521). The pooled analysis demonstrated no statistically significant difference in all-cause mortality between the MMAE and control groups (RR 0.72, 95% CI 0.37–1.43), with moderate heterogeneity observed among studies (I² = 42.8%) (Fig. 5**)**.

Mortality events were low in both groups (46/1,237 in the MMAE group vs. 66/1,284 in the control group). The overall confidence interval crossed unity, indicating no clear benefit or harm associated with MMAE. Given the low event rates and wide confidence intervals, this analysis is likely underpowered for small mortality differences.

##### Sensitivity analysis

Leave-one-out sensitivity analysis (excluding Lam et al., which reported no deaths in either arm) produced pooled RRs ranging from 0.63 to 0.84, with point estimates consistently below 1 but all confidence intervals crossing unity. Heterogeneity ranged from 45% to 56%, indicating that no single study materially altered the pooled result (Supplementary Figure S5).

#### Severe adverse events

**Fig. 6:**
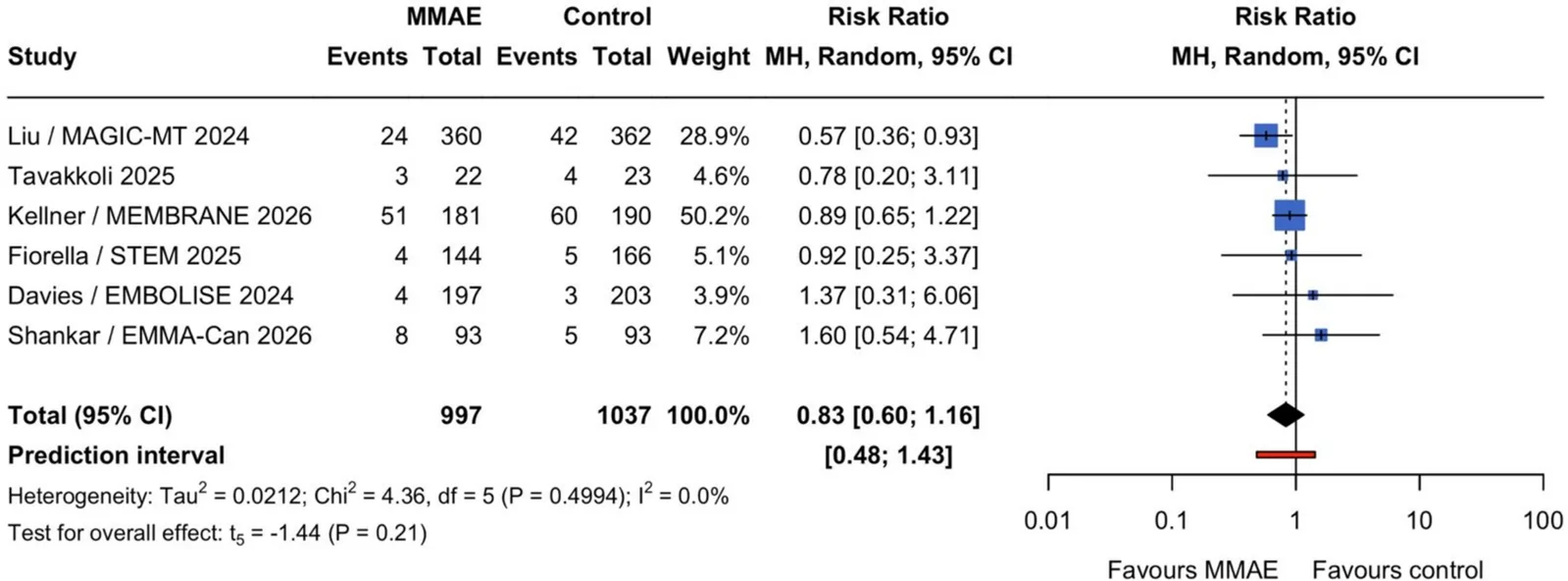
Forest plot of severe adverse events (study-defined composite) comparing MMAE versus standard management

Severe adverse events were reported in six RCTs (*n* = 2,034; 997 MMAE; 1,037 control) using varying definitions, including serious adverse events, procedural complications, stroke, and composite safety outcomes such as disabling stroke or death. Pooled analysis showed no statistically significant difference between MMAE and standard management (RR 0.83, 95% CI 0.60–1.16), and no statistical heterogeneity was observed (I² = 0%) (Fig. 6).

These findings should be interpreted cautiously because outcome definitions varied across trials and some composite endpoints combined events of differing clinical severity. In addition, the relatively small number of events limits the precision of the estimate. As a result, the lack of a statistically significant difference should not be interpreted as evidence of equivalent safety.

#### Radiological outcomes

Radiological outcomes were reported in three studies with differing outcome measures and follow-up timepoints. Ng et al. [29] reported greater haematoma volume resorption at 3 months in the MMAE group than in the control group (mean 52.6 ± 24.9 vs. 35.1 ± 21.0 mL). Similarly, Lam et al. [30] reported reduced residual haematoma thickness in the MMAE group at both 6 weeks (mean 6.56 vs. 9.2 mm) and 3 months (mean 2.14 vs. 3.76 mm). Shankar et al. [22] reported radiographic recurrence at 90 days in 13/93 patients in the MMAE group compared with 46/93 in the control group. However, due to inconsistent outcome measures, incomplete reporting of standard deviations, and differing follow-up intervals, pooled analysis was not performed.

#### Functional outcomes

Functional outcomes were inconsistently defined. Lam et al. [30] reported more favourable functional outcomes (mRS 0–1) in the MMAE group at 3 months compared with the control group (16/16 vs. 10/19). In contrast, Davies et al. [17] reported functional deterioration in 21/177 patients in the MMAE group and 18/184 in the control group. Shotar et al. [33] reported disability (mRS ≥ 4), although event data were not available for quantitative comparison. Owing to differences in outcome definitions and inconsistent reporting, a meta-analysis of functional outcomes was not performed.

The heterogeneity observed across studies likely reflects both clinical and methodological differences. These include variation in whether MMAE was used as a standalone or adjunctive treatment, variation in the use of embolic agents, differences in comparator groups, and heterogeneity in follow-up durations. These factors limit the interpretability and generalisability of pooled estimates and underscore the need for more standardised trial designs in future research.

### Quality assessment

Detailed risk-of-bias assessments are provided in Supplementary Appendix 2.

**Fig. 7:**
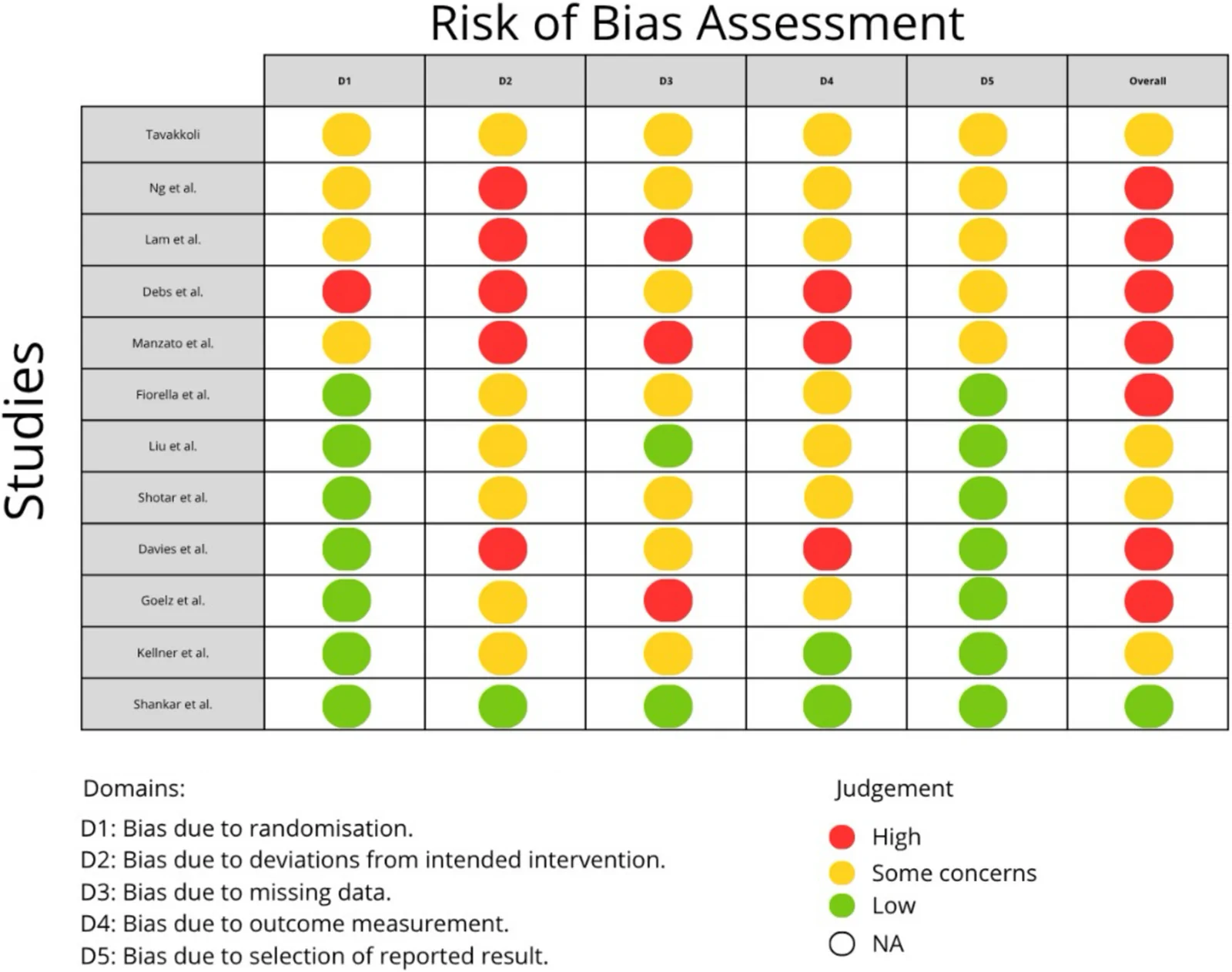
Summary of risk of bias assessment

The risk of bias assessment across the included RCTs (Fig. 7) demonstrated variability in methodological quality. Seven trials were judged to be at high overall risk of bias, four had some concerns, and one was judged to be at low overall risk. The principal methodological concerns related to deviations from intended interventions (D2), missing outcome data (D3), and measurement of the outcome (D4), particularly in open-label trials and studies with incomplete follow-up. These limitations were considered when assessing the certainty of evidence.

**Fig. 8:**
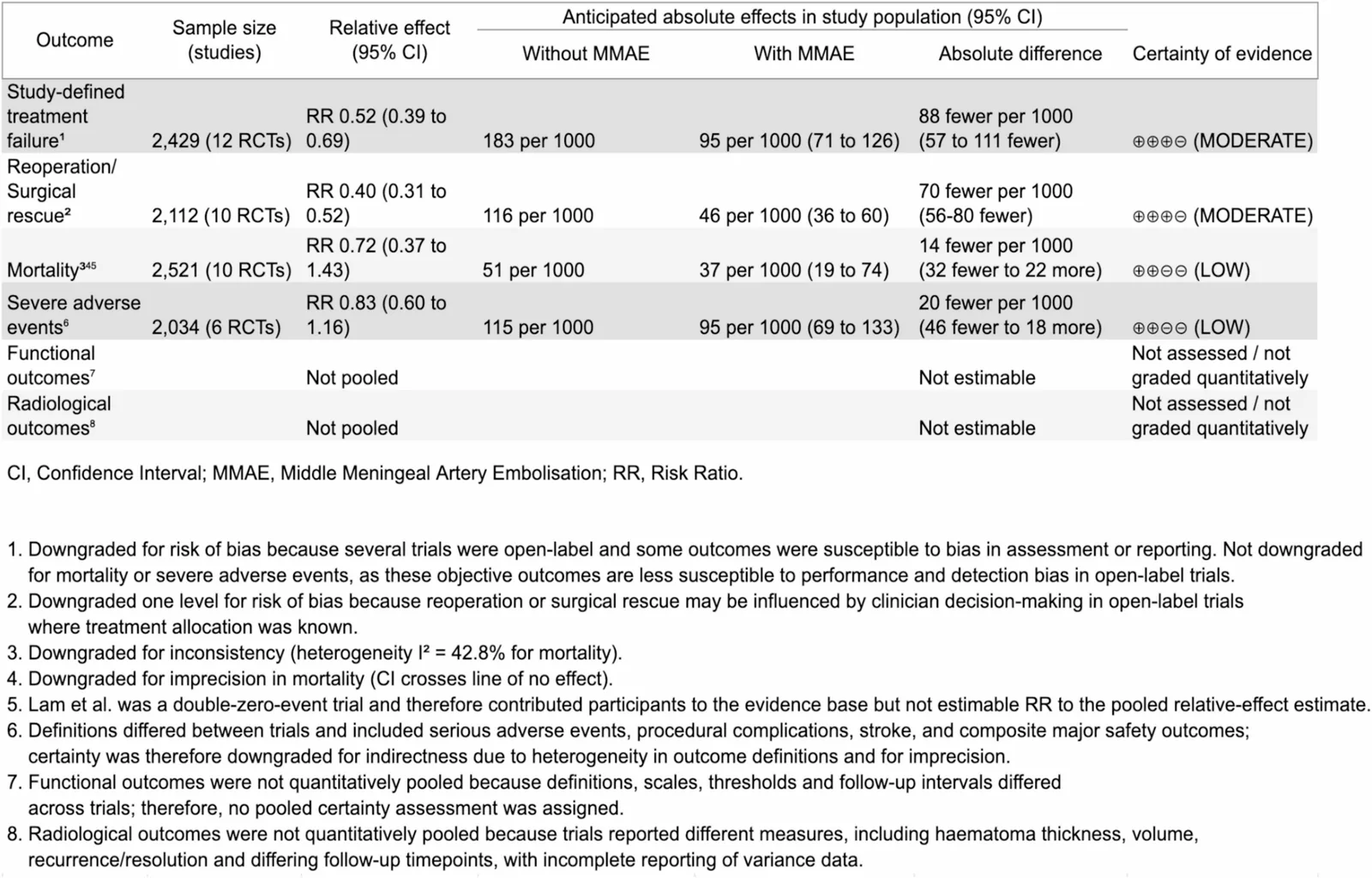
Summary of findings using GRADE framework

According to the GRADE framework (Fig. 8), the certainty of evidence for study-defined treatment failure was moderate, with MMAE reducing treatment failure compared with standard management (RR 0.52, 95% CI 0.39 to 0.69), corresponding to 88 fewer events per 1000 patients. The certainty of evidence for reoperation/surgical rescue was also moderate (RR 0.40, 95% CI 0.31 to 0.52), corresponding to 70 fewer reoperations/ surgical rescue events per 1000 patients. For all-cause mortality, the certainty of evidence was low (RR 0.72, 95% CI 0.37–1.43), with the confidence interval including both potential benefit and harm. Severe adverse events were also rated as low-certainty evidence (RR 0.83, 95% CI 0.60–1.16), owing to imprecision and variation in outcome definitions across trials. Functional and radiological outcomes were not quantitatively pooled because of heterogeneity in outcome definitions, assessment methods, and follow-up intervals. Therefore, no quantitative certainty rating was assigned for these outcomes.

## Discussion

This SRMA of 12 RCTs (primary analysis *n* = 2,429) showed that MMAE significantly reduced study-defined treatment failure (9.1% vs. 18.3%; RR 0.52, 95% CI 0.39–0.69; ARR 8.8%; NNT ~ 12) and reoperation or surgical rescue (RR 0.40, 95% CI 0.31–0.52), with no clear difference in all-cause mortality (RR 0.72, 95% CI 0.37–1.43), or severe adverse events (RR 0.83, 95% CI 0.60–1.16), both low-certainty. MMAE reduced treatment failure in surgery-only trials (RR 0.41, 95% CI 0.23–0.76; I² = 38.1%) and mixed management trials (RR 0.55, 95% CI 0.37–0.81; I² = 0%), without a significant subgroup difference (*p* = 0.317), providing no clear evidence that treatment effect differed according to comparator strategy. These findings remain exploratory. Sensitivity analyses also supported the robustness of the primary result, with pooled estimates remaining statistically significant in favour of MMAE throughout.

A source of clinical heterogeneity was variation in trial-specific definitions of treatment failure, which we retained rather than imposing a uniform composite definition – a choice that may limit direct comparability across studies but avoids retrospective reclassification. These definitions included recurrence, progression, reintervention, reoperation, surgical rescue, and other trial-defined endpoints, so reoperation/surgical rescue was analysed separately as a more clinically consistent outcome.

TSA further supported efficacy: for both study-defined treatment failure and reoperation/surgical rescue, the cumulative Z-curves crossed the sequential monitoring boundaries for benefit despite only 47% and 39% of the respective RIS having accrued. These findings provide sequential evidence of benefit under the prespecified TSA assumptions, though further trials are needed to clarify optimal pathways, embolic techniques, and safety. Our findings are broadly consistent with recent RCT-focused meta-analyses: Gonçalves et al. [21] (adjunctive MMAE plus surgery) similarly reported reductions in treatment failure (RR 0.48, 95% CI 0.33–0.71) and reoperation (RR 0.33, 95% CI 0.21–0.52), with their TSA indicating the RIS had been reached for both outcomes; Elgendy et al. [20] reported reduced recurrence (RR 0.52, 95% CI 0.37–0.73) and surgical rescue (RR 0.36, 95% CI 0.25–0.53), with moderate certainty and crossed TSA boundaries for both. The apparent discrepancy with Gonçalves et al. [21], who reported RIS values of 702 for treatment failure and 214 for reoperation, should be interpreted in the context of differences in trial eligibility and outcome definitions, and TSA assumptions. Although both analyses used a 5% significance level and 80% power, the RIS is also sensitive to the anticipated relative risk reduction, control-group event rate, statistical power, significance level, and adjustment for between-study heterogeneity. Differences in these parameters may therefore contribute to different RIS conclusions. Reaching the RIS does not itself confer high GRADE certainty: our review retained moderate certainty because of methodological limitations identified through RoB 2 assessment, whereas Gonçalves et al. rated the efficacy evidence as high certainty.

The observed benefits are biologically plausible, as MMAE targets the vascular supply of the pathological subdural membranes implicated in cSDH persistence and recurrence [34, 35], potentially complementing surgical evacuation by addressing membrane vascularity associated with ongoing haematoma formation.

A strength of this review is its restriction to RCTs, limiting confounding and selection bias, though at a cost to breadth given that much earlier MMAE literature was observational. The addition of three recent RCTs, separate analysis of reoperation/surgical rescue, TSA, and updated RoB 2/GRADE assessments together provides a more current appraisal of the randomised evidence.

Another limitation concerns how MMAE was used across trials. Ideally, standalone and adjunctive MMAE would be analysed separately, as these represent distinct clinical treatment strategies. However, this was not consistently feasible since several trials incorporated mixed treatment pathways without reporting outcomes separately by strategy. We evaluated MMAE as reported in the original trials rather than reclassifying patients into subgroups not uniformly defined across studies. The objective was to evaluate the effectiveness of MMAE-based treatment strategies within cSDH management rather than compare specific treatment pathways. Restricting the analysis to adjunctive-MMAE trials would have excluded several large mixed-pathway RCTs and reduced the evidence base. Although this approach may reduce the clinical granularity of the findings, it preserves consistency with the original trial designs and synthesises the randomised evidence currently available.

Methodological quality varied: seven studies were at high overall risk of bias, four had some concerns, and one was low overall risk, with principal concerns relating to deviations from intended interventions, missing outcome data, and outcome measurement – particularly in open-label trials and studies with incomplete follow-up. Certainty was moderate for study-defined treatment failure and reoperation/surgical rescue, but low for all-cause mortality and severe adverse events. Manzato et al. [32] randomised haematomas rather than individual patients, introducing possible within-patient correlation from bilateral haematomas. However, exclusion of this study in the leave-one-out analysis did not materially change the pooled estimate (RR 0.53, 95% CI 0.39–0.71). Tavakkoli et al. [28] was an unreviewed preprint at the time of analysis. Although its exclusion did not materially alter the treatment-failure estimate, non-peer-reviewed evidence may carry unverified methodological limitations or reporting bias, which should be weighed alongside the RoB 2 and GRADE assessments. Lastly, variability in outcome definitions prevented quantitative pooling of other secondary endpoints such as functional status and radiological resolution, underscoring the need for standardised trial designs in future research.

Current randomised evidence suggests that MMAE-based treatment reduces study-defined treatment failure and reoperation/surgical rescue, although uncertainty remains regarding the optimal patient population, treatment pathways, timing, and embolic techniques. Further well-conducted studies are required to strengthen this evidence and inform clinical guidelines.

## Conclusion

MMAE-based treatment strategies reduce study-defined treatment failure and reoperation/surgical rescue in patients with cSDH compared with standard management, while no clear difference has been demonstrated in all-cause mortality or severe adverse events. Variation in trial design, treatment pathways, outcome definitions, and follow-up durations limits certainty regarding optimal implementation. Further well-designed RCTs with standardised efficacy and safety outcomes are needed to define the optimal role and implementation of MMAE within multidisciplinary cSDH care.

Abbreviations: cSDH, chronic subdural haematoma; ITT, intention-to-treat; mITT, modified intention-to-treat; MMAE, middle meningeal artery embolisation; n-BCA, n-butyl cyanoacrylate; NR, not reported; NSMM, nonsurgical medical management; PHIL, precipitating hydrophobic injectable liquid; PVA, polyvinyl alcohol; RCT, randomised controlled trial; SDH, subdural haematoma; SOC, standard of care; TAGM, tris-acryl gelatin microspheres; TRUFILL n-BCA, TRUFILL n-butyl cyanoacrylate liquid embolic system.

## Electronic Supplementary Material

Below is the link to the electronic supplementary material.

Supplementary Material 1

Supplementary Material 2

## Data Availability

No datasets were generated or analysed during the current study.
