# Supplementary material for: Efficacy and safety of middle meningeal artery embolisation for chronic subdural haematoma: A systematic review and meta-analysis of randomised controlled trials
Source: Neurosurg Rev. 2026 Nov 11;49(1):646. doi: 10.1007/s10143-026-04568-4 (PMC13754966; doi:10.1007/s10143-026-04568-4)

SUPPLEMENTARY MATERIAL

Efficacy and Safety of Middle Meningeal Artery Embolisation for Chronic Subdural Haematoma: A Systematic Review and Meta-analysis of Randomised Controlled Trials

TABLE OF CONTENTS

- **Supplementary Appendix 1. Search Strategies**
- **Supplementary Table S1. Trial-specific definitions of study-defined treatment failure used in the meta-analysis**
- **Supplementary Figure S1. Drapery plot for study-defined treatment failure**
- **Supplementary Figure S2. PRISMA flow diagram**
- **Supplementary Figure S3. Trial sequential analysis for study-defined treatment failure**
- **Supplementary Figure S4. Trial sequential analysis for reoperation/surgical rescue**
- **Supplementary Figure S5. Leave-one-out sensitivity analysis for all-cause mortality**

Appendix 1. Search strategies

PUBMED 205

("Hematoma, Subdural, Chronic"[Mesh] OR "chronic subdural hematoma"[Title/Abstract] OR "chronic subdural haematoma"[Title/Abstract] OR cSDH[Title/Abstract])

AND

("middle meningeal artery"[Title/Abstract] OR "middle meningeal arteries"[Title/Abstract] OR MMA[Title/Abstract])

AND

(emboliz*[Title/Abstract] OR embolis*[Title/Abstract])

AND

(randomized controlled trial[pt] OR controlled clinical trial[pt] OR randomized[tiab] OR randomised[tiab] OR randomly[tiab] OR placebo[tiab] OR trial[ti] OR groups[tiab])

NOT

(animals[mh] NOT humans[mh])

Cochrane 86

1. MeSH descriptor: [Hematoma, Subdural, Chronic] explode all trees
2. "chronic subdural hematoma":ti,ab,kw OR "chronic subdural haematoma":ti,ab,kw OR cSDH:ti,ab,kw
3. #1 OR #2
4. "middle meningeal artery":ti,ab,kw OR "middle meningeal arteries":ti,ab,kw OR MMA:ti,ab,kw
5. emboliz*:ti,ab,kw OR embolis*:ti,ab,kw
6. #4 AND #5
7. #3 AND #6

Web of Science 167

TS=(

("chronic subdural hematoma" OR "chronic subdural haematoma" OR cSDH)

AND

("middle meningeal artery" OR "middle meningeal arteries" OR MMA)

AND

(emboliz* OR embolis*)

AND

(random* OR trial)

)

Ovid (MEDLINE and EMBASE) 144

1. Hematoma, Subdural, Chronic/
2. (chronic subdural hematoma or chronic subdural haematoma or cSDH).ti,ab.
3. 1 or 2
4. ((middle adj2 meningeal adj2 arter$) or MMA).ti,ab.
5. (emboliz$ or embolis$).ti,ab.
6. 4 and 5
7. 3 and 6
8. randomized controlled trial.pt.
9. controlled clinical trial.pt.
10. random*.ti,ab.
11. trial.ti.
12. 8 or 9 or 10 or 11
13. exp animals/ not humans.sh.
14. 7 and 12
15. 14 not 13

Table S1. Trial-specific definitions of study-defined treatment failure used in the meta-analysis

Study	Outcome entered as treatment failure
Tavakkoli 2025	Failure of treatment success
Ng 2020	Surgical rescue
Lam 2023	Symptomatic recurrence requiring repeat evacuation
Debs 2024	Reintervention
Manzato 2025	Recurrence requiring repeat surgery
Fiorella / STEM 2024	Primary efficacy composite
Liu / MAGIC-MT 2024	Symptomatic recurrence/progression
Shotar / EMPROTECT 2025	cSDH recurrence
Davies / EMBOLISE 2024	Reoperation/ indication for surgery
Shankar / EMMA-Can 2026	Symptomatic cSDH recurrence
Goelz / G-MEMBRANE 2026	Primary recurrence composite
Kellner / MEMBRANE 2026	Residual/reaccumulated >10 mm or surgery

Figure S1. Drapery plot for study-defined treatment failure

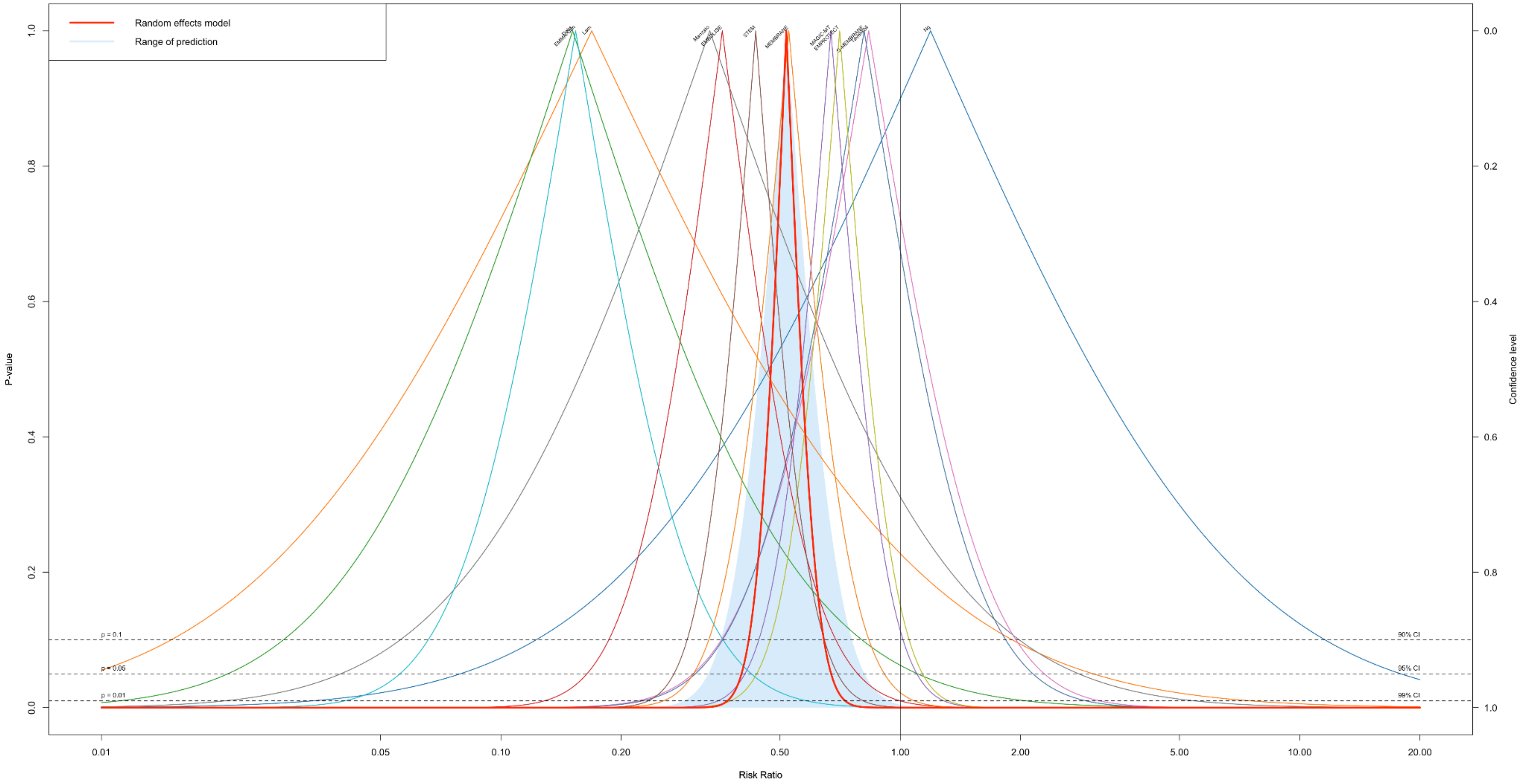

Figure S2. PRISMA flow diagram

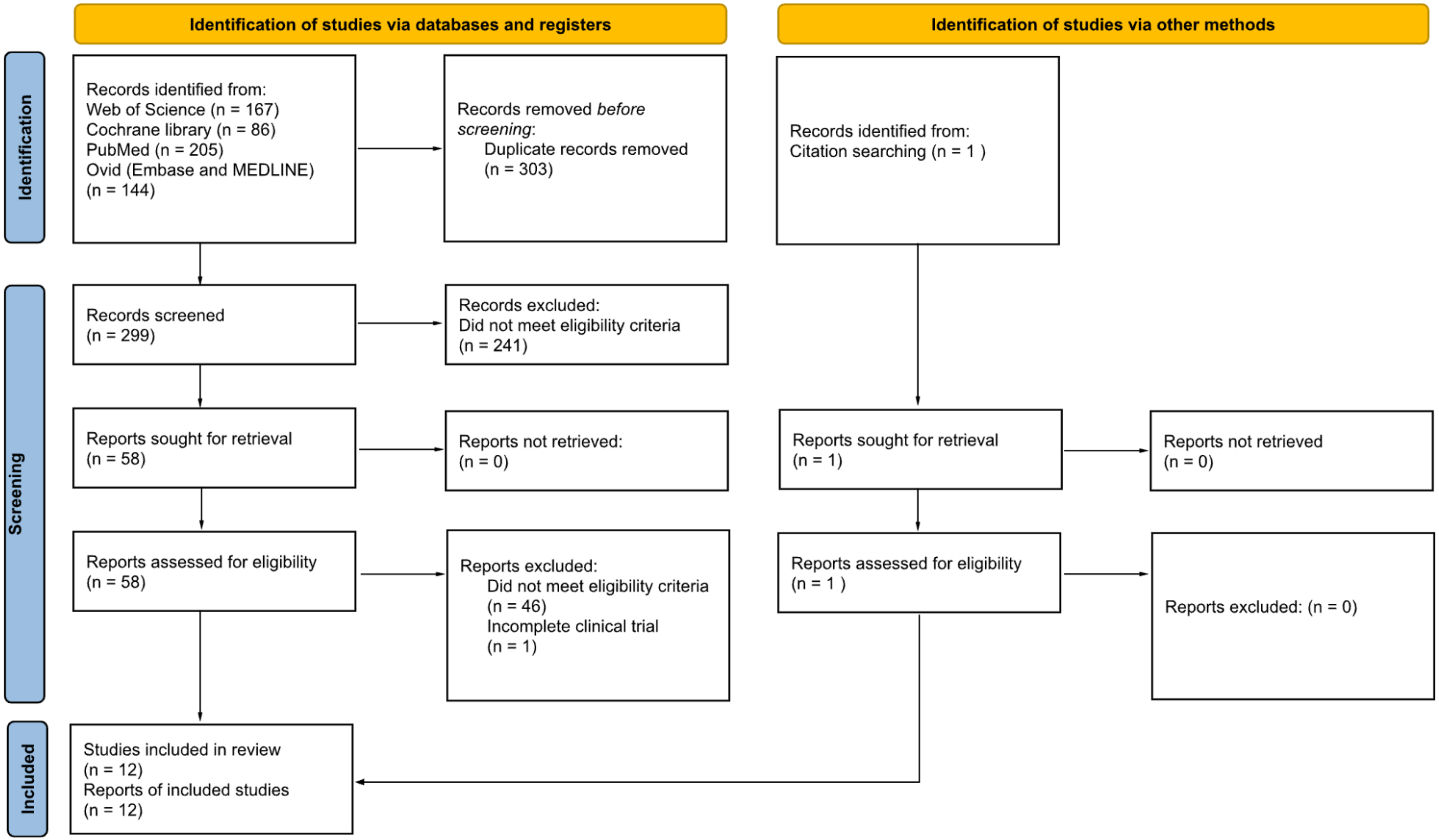

Figure S3. Trial sequential analysis for study-defined treatment failure

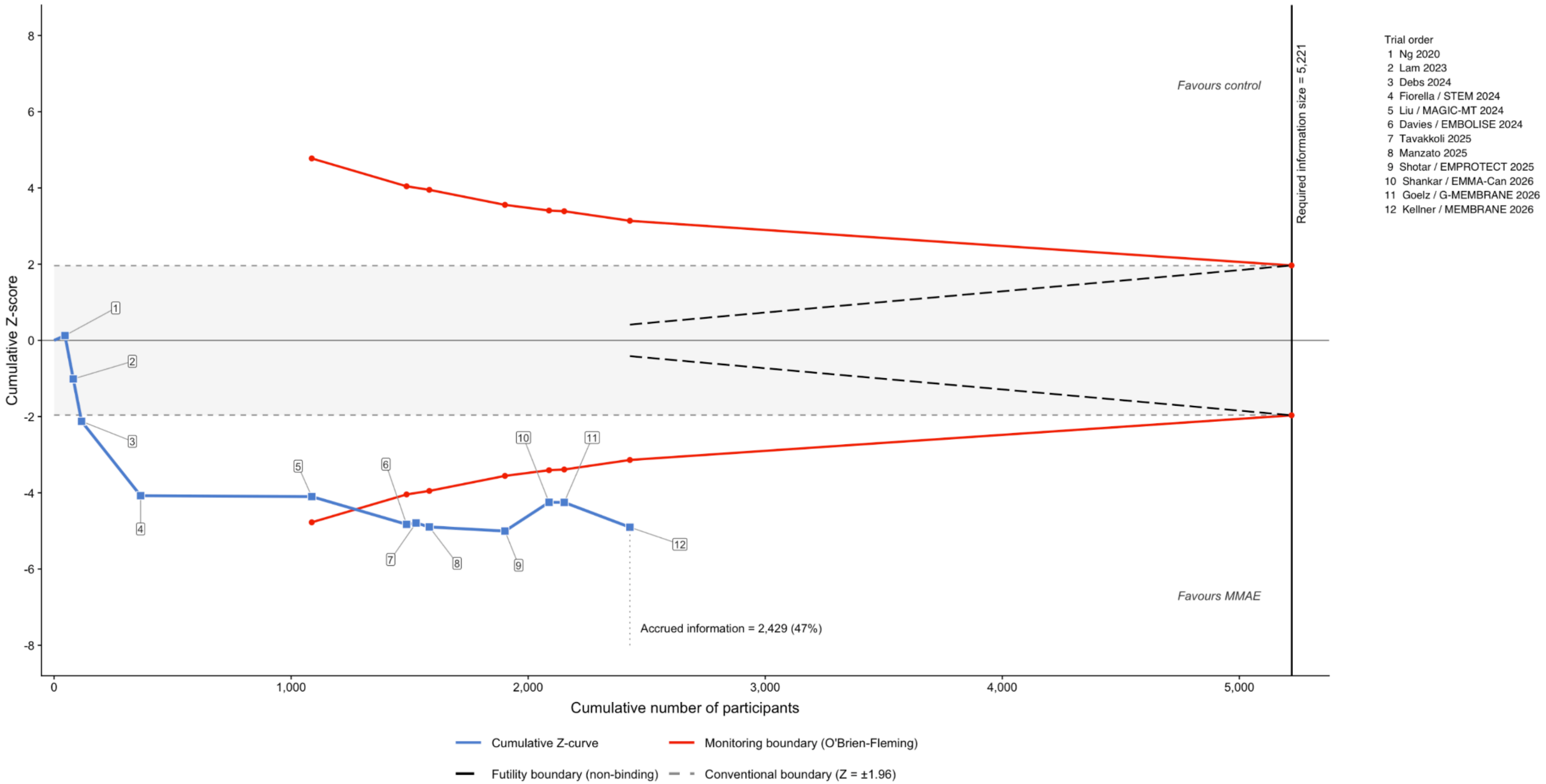

Two-sided α = 5%, power 80%, anticipated RRR 20%, control event rate 18.3%; information size adjusted for diversity (D^2 = 34%).
Z-curve: Mantel-Haenszel common-effect while DerSimonian-Laird τ^2 = 0 (trials 1–9); DerSimonian-Laird random-effects with Hartung-Knapp-Sidik-Jonkman variance thereafter (trials 10–12).
Non-binding futility boundaries (O'Brien-Fleming β-spending) are defined from trial 12 (47% of the RIS); at earlier looks they would fall below Z = 0, where RTSA does not compute them.

Figure S4. Trial sequential analysis for reoperation/ surgical rescue

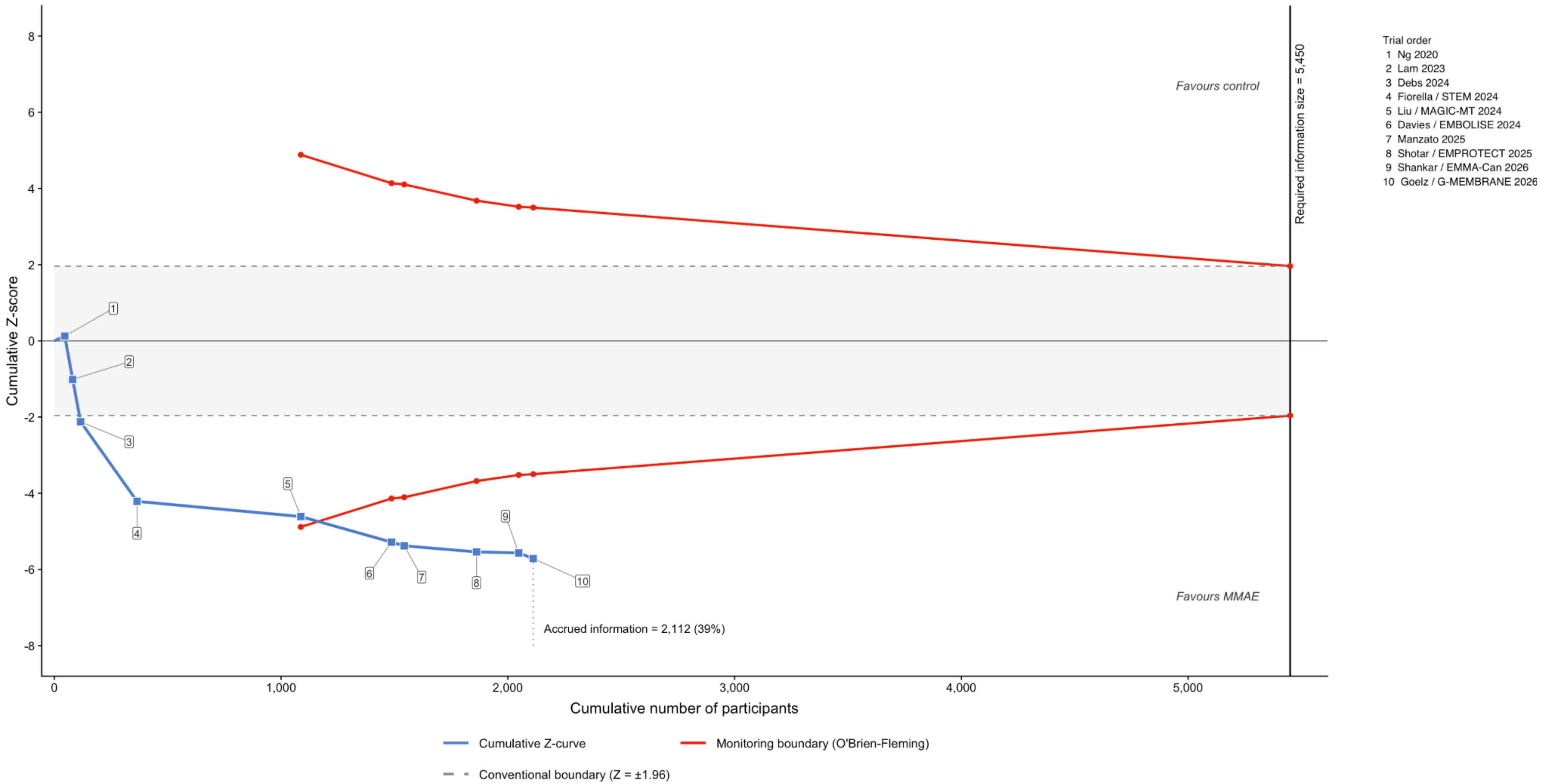

Two-sided α = 5%, power 80%, anticipated RRR 20%, control event rate 11.6%; D^2 = 0%, so no heterogeneity adjustment to the information size.
Z-curve: Mantel-Haenszel common-effect statistic at every look (DerSimonian-Laird τ^2 = 0 throughout).
No futility boundary is shown: at the last look (39% of the RIS) the non-binding futility boundary would still fall below Z = 0, so RTSA does not define one.

Figure S5. Leave-one-out sensitivity analysis for all-cause mortality

Study omitted	RR [95% CI]	Tau²	I²	P value
Tavakkoli 2025	0.82 [0.42; 1.60]	0.24	46%	0.51
Liu / MAGIC-MT 2024	0.82 [0.40; 1.68]	0.26	48%	0.54
Kellner / MEMBRANE 2026	0.84 [0.39; 1.82]	0.28	46%	0.60
Ng 2020	0.73 [0.35; 1.56]	0.31	49%	0.36
Manzato 2025	0.75 [0.34; 1.68]	0.39	56%	0.43
Shotar / EMPROTECT 2025	0.73 [0.31; 1.72]	0.50	56%	0.41
Fiorella / STEM 2025	0.63 [0.29; 1.37]	0.29	46%	0.20
Davies / EMBOLISE 2024	0.63 [0.30; 1.31]	0.25	45%	0.18
Shankar / EMMA-Can 2026	0.66 [0.34; 1.30]	0.29	48%	0.19
**All studies included**	**0.72 [0.37; 1.43]**			

All studies: Tau² = 0.28; I² = 43%; test for overall effect P = 0.30

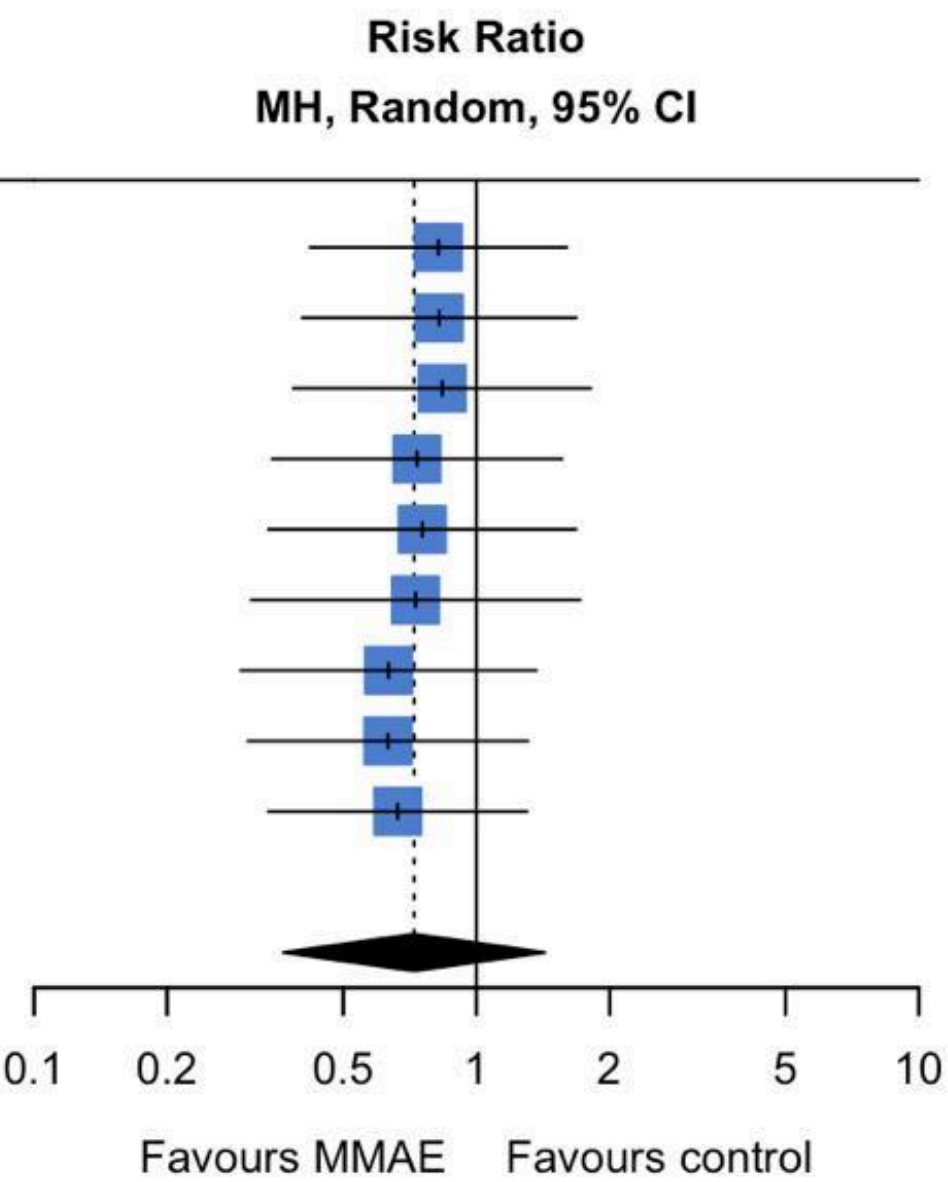

Supplement: Supplementary file 1 [file 10143_2026_4568_MOESM1_ESM.pdf]
